# Fabrication of nitrogen doped TiO_2_/Fe_2_O_3_ nanostructures for photocatalytic oxidation of methanol based wastewater

**DOI:** 10.1038/s41598-023-31625-5

**Published:** 2023-03-17

**Authors:** Mai Mersal, Abdallah F. Zedan, Gehad G. Mohamed, Gamal K. Hassan

**Affiliations:** 1 National Institute of Laser Enhanced Science, Cairo University, Giza, 12613 Egypt; 2 Egypt Nanotechnology Center (EGNC), Cairo University, El-Sheikh Zayed, Giza Egypt; 3 Department of Chemistry, Faculty of Science, Cairo University, Giza, 12613 Egypt; 4 Nanoscience Department, Basic and Applied Sciences Institute, Egypt-Japan University of Science and Technology, New Borg El Arab, Alexandria 21934 Egypt; 5 Water Pollution Research Department, National Research Centre, 33El-Bohouth St. (Former El-Tahrir St.), Dokki, Giza, 12622 Egypt

**Keywords:** Environmental sciences, Chemistry, Materials science, Nanoscience and technology

## Abstract

An important industrial process that often occurs on the surface of a heterogeneous catalyst using thermochemical or photochemical could help in the oxidation of methanol-based wastewater to formaldehyde. Titania-based photocatalysts have drawn a lot of interest from scientists because they are a reliable and affordable catalyst material for photocatalytic oxidation processes in the presence of light energy. In this study, a straight-forward hydrothermal method for producing n-TiO_2_@α-Fe_2_O_3_ composite photocatalysts and hematite (α-Fe_2_O_3_) nanocubes has been done. By adjusting the ratio of n-TiO_2_ in the prepared composite photocatalysts, the enhancing influence of the nitrogen-doped titania on the photocatalytic characteristics of the prepared materials was investigated. The prepared materials were thoroughly characterized using common physiochemical methods, such as transmission electron microscope (TEM), scanning electron microscope (SEM), X-ray diffraction (XRD), energy dispersive X-ray (EDX), X-ray photoelectrons spectroscopy (XPS), physisorption (BET), and others, in order to learn more about the structure The results obtained showed that nitrogen-doped titania outperforms non-doped titania for methanol photooxidation. The addition of nitrogen-doped titania to their surfaces resulted in an even greater improvement in the photooxidation rates of the methanol coupled with hematite. The photooxidation of methanol in the aqueous solution to simulate its concentration in the wastewater has been occurred. After 3 h, the four weight percent of n-TiO_2_@α-Fe_2_O_3_ photocatalyst showed the highest rate of HCHO production.

## Introduction

The removal of water pollutants such as aromatics, petroleum-based materials, chlorinated hydrocarbons, pesticides, insecticides, volatile organic compounds (VOC), dyes, and other organic materials using advanced oxidation processes (AOPs) is an environmentally friendly method^1^. They have short lives since they primarily rely on the production of reactive oxygen species like hydroxyl radicals. As a result, they quickly and actively interact with a variety of chemical species, many of which are challenging to degrade^1^. Moreover, AOPs, have been used for the treatment of many types of wastewaters as a promising technology that can effectively reduce the concentrations of recalcitrant organic contaminants and the created oxidation products, such as carbon dioxide, water, and biodegradable organics, are thermodynamically stable, making them superior to other traditional approaches^2^. AOPs contain the photocatalysis process, which is crucial for a photocatalyst to harvest sunlight^3,4^. Then, in the presence of various solar spectrum ranges, these photocatalysts have been used successfully to address issues connected to environmental pollution and energy crises^3,4^.

Numerous studies like^5^ have been conducted on photocatalytic oxidation of organic compounds to CO_2_. Recently, many novel nanoparticles have been fabricated for environmental applications^6^. The most popular nano-catalyst and photo-catalyst in the same time is titanium (TiO_2_) because it is readily available and stable under a variety of reaction settings^7^. Being exclusively triggered by UV light is one of TiO_2_'s biggest drawbacks. It is desirable to have photocatalysts that can use solar radiation and visible light with less energy. With a band gap energy of 2.8 eV, tungsten oxide (WO_3_) can be photoactivated by blue light (500 nm) and is a suitable substitute for TiO_2_ as a photocatalyst. To raise catalytic rates, as is the case with all photocatalysts, it is necessary to enhance the charge separation of electrons and holes. Light with enough energy is absorbed by metal oxide photocatalysts like WO_3_^8,9^ and TiO_2_ to cause band gap excitation and the generation of reactive electrons (e^−^) and holes (h^+^), which are in charge of catalytic reactions^10^.

Formaldehyde (HCHO, 60%) and methyl formate (MF), respectively, were produced by highly selective MeOH photooxidation catalysts supported on WO_3_ and SiO_2_-supported Au NPs. This demonstrated that the two separate oxidation products were produced by two distinct photocatalytic processes, namely band gap excitation (WO_3_) and surface plasmon resonance (SPR, Au NPs). Due to more effective electron and hole separation when coupled with Au NPs, WO_3_ demonstrated higher rates of MeOH oxidation than it did when used alone. However, due to increased oxidation activity of the mixed WO_3_-Au catalyst at higher Au NP loadings, selective oxidation of MeOH decreased at the expense of increasing CO_2_ production^10^.

Methanol is considered an organic compounds and present in many types of wastewater such as landfill leachate^11,12^. Methanol is frequently used as a hole scavenger in photocatalysis and is also researched as a model system for heterogeneous photocatalysis to make formaldehyde. When taking into account the following factors, photocatalytic oxidation of methanol-based wastewater to formaldehyde may be more promising than commercial systems for producing formaldehyde using thermal catalytic methanol oxidation. (1) lower reaction temperature and pressure (often operated at standard conditions); (2) cheaper energy source (such solar light); and (3) simple to construct reactive system^8^. Bi_2_WO_6_, Bi_2_MoO_6_, BiOBr, and BiVO_4_ were used in photocatalytic methanol oxidation experiments with four distinct bismuth-based semiconductors (BBS), with changing system temperature, bubbling speed, catalyst dosage, and initial methanol concentration in the wastewater. It was revealed that each of these system characteristics increased the formaldehyde generation rate for all photocatalysts. Bi_2_WO_6_ was reported to have the greatest formaldehyde production rate (0.081 mM/h) among all four BBS^13^.

During photocatalytic oxidation (PCO) of fluidized beds at different titania-based catalysts, experiments were conducted to assess the rates of methanol elimination and catalyst elutriation^14^. The research created elutriation-resistant catalysts that were coated with TiO_2_/Al_2_O_3_ or precipitated from solution (p-TiO_2_) and compared them to Degussa P-25 TiO_2_. The rate at which Degussa P-25 TiO_2_ eluted was two orders of magnitude higher than that of p-TiO_2_ and TiO_2_/Al_2_O_3_, despite the fact that it successfully oxidized methanol^14^. TiO_2_/Al_2_O_3_ considerably eliminated more methanol than P-25 and p-TiO_2_ (being the least active). Greater PCO rates were produced by fluidized beds than by packed P-25 and TiO_2_/Al_2_O_3_ beds^14^. The performance of the P-25 fluidized bed was enhanced by fluidization enhancers including vibration and the addition of a static mixer, but the efficacy of TiO_2_/Al_2_O_3_ or p-TiO_2_ was unaffected^10^. The optimal TiO_2_ loading for TiO_2_/Al_2_O_3_ was 30 wt%, and the activity of TiO_2_/Al_2_O_3_ decreased with rising calcination temperature^14^.

Composites of titanium dioxide and single-walled carbon nanotubes (TiO_2_/SWNT) were created and studied using a variety of techniques, including scanning and transmission electron microscopy combined with energy dispersive X-ray spectroscopy, Raman, UV–visible, and zeta-potential measurements. The photo-oxidation of methanol in sulfuric acid as the supporting electrolyte was used to examine the photocatalytic activity of TiO_2_ and the TiO_2_/SWNT composite^15^. According to the findings, TiO_2_/SWNT combination has higher photocatalytic activity than TiO_2_ alone^15^.

By using coumarin and coumarin-3-carboxylic acid as fluorescent probes, the oxidation reaction processes of organic additives, such as alcohols (MeOH and EtOH), and different inorganic ions in photocatalysis were clarified^16^. The overall plan for using coumarin to oxidise and probe OH radicals was put forth. According to research, organic molecules should undergo photocatalytic oxidation by the action of holes trapped on the TiO_2_ surface, but inorganic ions were found to undergo photocatalytic oxidation through OH radicals^16^. The quantum efficiency of the CO_2_ generation by MeOH oxidation was almost ten times greater than that of the OH radical formation, which supports this^16^.

Aerated carbon-doped TiO_2_ (C-TiO_2_) aqueous suspensions exhibit visible photolysis, which triggers the oxidation of methanol-based wastewater to formaldehyde^17^. When there is an excess of H_2_O_2_ or catalase present, the rate of HCHO production roughly doubles and increases with CH_3_OH or C-TiO_2_ concentration^17^. The method entails surface trapped holes oxidizing CH_3_OH, however, these holes are less energetic than those produced by UV photolysis of undoped TiO_2_. O_2_ is converted to H_2_O_2_ by the C-TiO_2_ electrons^17^.

Due to its use in photocatalytic oxidation under UV irradiation, TiO_2_ photocatalytic particles have been extensively researched^18^. Strong oxidizing power^19–21^ and physical and chemical stability^22^ are two characteristics of TiO_2_ that make it effective in this application. Due to the anatase TiO_2_'s 3.2 eV bandgap energy, the photocatalytic characteristics of TiO_2_ can be produced under UV light^23^. Due to changes in characteristics of the selected catalyst, TiO_2_ nanoparticles made using various methods have demonstrated varying photocatalytic efficiency^23–25^. Integrating carbon nanotubes with TiO_2_ nanoparticles should lead to changes in morphology and enhanced properties, in particular electrical conductivity of TiO_2_, due to their distinctive properties, which include high surface area, high electrical conductivity, and significant mechanical strength^26^.

Finding novel photocatalyst materials has been a focus of research ever since Honda and Fujishima discovered in 1972 that TiO_2_ can be used for water splitting through electrochemical photolysis. Due to their potential for use in electrical, (photo) catalytic, photovoltaic, and energy-storage applications, mixed metal oxides have gained interest^27,28^. Doping several transition metals as Ru^3+^, Fe^3+^, Cr^3+^, and Cu^2+^ into TiO_2_ has been reported to increase the photocatalytic activity and produce an optical absorption shift to visible light^29^. This offers highly mobile photoexcited electrons that are useful for photocatalytic reactions. Metal oxides with d^10^ core metal ions and covalent metal ions as secondary ions are predicted to be efficient photocatalysts because they produce deformed metal–oxygen octahedral/tetrahedral coordination^30^. The general splitting of water to produce H_2_ and O_2_ was found to be a stable photocatalyst using 35zeolite membrane/RuO_2_, RuO_2_/BaTi_4_O_9_, and RuO_2_-dispersed Zn_2_GeO_4_^31^.

The test reaction for determining the mesoporous RuO_2_-TiO_2_ nanocomposites' photocatalytic activity under UV and visible light is the photocatalytic methanol oxidation to HCHO^32^. The amount and degree of RuO_2_ particle loading onto the TiO_2_ have a significant impact on the photooxidation of methanol, indicating the RuO_2_ nanoparticles' unique influence on this photocatalytic reaction under visible light. A minor quantity of Ru^4+^ being incorporated into the anatase lattice is what is responsible for the visible-light photocatalytic action^32^.

Because methanol can be created from natural gas, coal, and biomass and be converted to a variety of industrially significant compounds, methanol has gained a lot of attention as a starting material for numerous chemicals. As a result, post-oil methods will expedite the production of methanol and replace petroleum-derived raw materials as starting materials in the chemical sector. Because the oxidation produces significant precursors to more valuable compounds, the (thermo) catalytic oxidation of methanol has been the subject of numerous investigations^33^. The creation of a titanium (IV) oxide (TiO_2_) photocatalyst for the purification of polluted air and water has received a lot of attention^34^. There haven't been many investigations into selective oxidation over TiO_2_ photocatalysts, however^35^, perhaps due to the fact that total oxidation predominates over TiO_2_ and the selectivity of intermediate products declines dramatically.

Researchers looked at the photocatalytic oxidation of methanol to formaldehyde using nanocrystalline TiO_2_ (Degussa P-25) photocatalyst calcined at various temperatures with varying ratios of anatase (A)-rutile (R) phase composition^36^. With the aid of the fluorescence approach and terephthalic acid as a probe molecule, the calcined nano-crystalline TiO_2_ was characterized and the generation of hydroxyl radicals during the reaction was determined^36^. By oxidizing methanol in the presence of direct sunshine, the photocatalytic activity of catalysts was assessed and compared to that of pure anatase TiO_2_^36^.

Methyl formate was produced with a high level of selectivity (91 mol%) by photocatalytic oxidation of methanol in air over titanium(IV) oxide particles in a flow-type reactor at room temperature without catalyst deactivation because to the deposition of intermediate(s)^37^. Methanol conversion improved as reaction temperature was raised up to 523 K while maintaining high selectivity^37^.

The process of photooxidation of methanol involves the production of highly oxidizing (COH) radicals by the oxidation of hydroxide ions that have been adsorbed on the surface of photocatalysts. These radicals then attack the molecules of pollutants that have been adsorbed. This first stage of HCHO production sets off a chain of degradation processes that eventually cause the contaminants to become mineralized^38^. As a result, a tried-and-true method for figuring out the absolute COH radical generation rate is based on watching how quickly the first stable oxidation product, HCHO, is forming after the COH radicals remove the a-H atom from methanol^39^. Additionally, it has been shown how TiO_2_ anatase, rutile, and brookite can photocatalytically selectively oxidize 4-methoxybenzyl alcohol to aldehyde in aqueous solutions^40^.

Despite due to its chemical and photostability, cheap cost, and availability, TiO_2_ is one of the most thoroughly explored photocatalysts^41–45^. In spite of this, its broad bandgap causes a high rate of photogenerated charge carrier recombination and poor activity in visible light (Eg). Making a heterostructure with a different, more restricted Eg visible active semiconductor is one approach to solving this issue^41–45^. It is essential for semiconductor photocatalysts to align their bands (CB and VB). Hematite Fe_2_O_3_ was chosen as a visible active photocatalyst (Eg = about 2 eV) and mixed with TiO_2_ to create the composite, which was then studied. The separation of photogenerated electron–hole pairs will be aided by the formation of an internal electric field and the alignment of TiO_2_ and Fe_2_O_3_'s Fermi energy levels. TiO_2_-Fe_2_O_3_ heterostructures have therefore been used in a variety of photocatalytic-based applications, such as a potent photoanode for water oxidation in photoelectrochemical cells (PEC), photoreduction of Cr (VI), and dye degradation^41–45^. As in the case of PEC applications, certain applications apply external bias to lower the recombination rate. Studying the various characteristics of Fe-doped TiO_2_^41–45^ is relevant considering these significant and future uses. In light of this, n-dopped titanium dioxide/Fe_2_O_3_ nanocomposite for photocatalytic applications was created. UV–visible, XRD, Raman, zeta-potential measurements, scanning and transmission electron microscopes, together with energy dispersive X-ray spectroscopy, were used to characterize the nanocomposite. The photooxidation of methanol in the aqueous solution to simulate its concentration in the wastewater was used to test the photocatalytic activity of the composite n-TiO_2_@α-Fe_2_O_3_ photocatalyst. According to the findings, the n-TiO_2_@α-Fe_2_O_3_ composite photocatalyst improves on n-TiO_2_ photocatalyst alone in terms of photocatalytic activity.

## Materials and methods

### Materials

The chemicals and reagents were bought from domestic or foreign vendors, and they were used directly after purchase without any further processing. The materials included ferric nitrate—nonahydrate (Fe(NO_3_)_3_⋅9H_2_O, 98%, SD Fine Chem. LTD), sodium hydroxide pellets (97%, ACROS Organics), glacial acetic acid (98%, CARLO ERBA chemicals), acetyl acetone (98%, LOBA Chemie), formaldehyde (37%, ROTH) and nitrogen-doped titanium dioxide (Company, China). Methanol (99.8%), titanium dioxide (99%), sodium chloride (98.5%), hydrochloric acid (37%), ammonium acetate (99.99%), potassium borohydride (98%) and polyvinyl pyrrolidone (PVP, 55.000 g/mol) were supplied from Sigma-Aldrich.

### Solvothermal synthesis of n-TiO_2_@α-Fe_2_O_3_ nanocubes photocatalyst

A two-step solvothermal process was used to fabricate n-TiO_2_@α-Fe_2_O_3_. First, by hydrothermally treating the metal salt under extremely alkaline circumstances, -Fe2O3 nanocubes were created. Aqueous solutions of 0.0247 mol of Fe(NO_3_)_3_.9H_2_O dissolved in 40 ml of water and 0.05 mol of NaOH/10 ml of water were created in a PTFE jar while being continuously stirred. The mixture was agitated for 30 min using a hotplate/stirrer after being sonicated for 20 min in an ultrasonic bath (35 kHz, Iranssonic, Elma, Germany) (Model 1000, Jenway, UK). The mixture was placed in a PTFE vessel, which was then moved into a chamber of a stainless steel autoclave, which was then sealed and heated to 160 °C for 24 h in an electric oven (Linn-High-Therm, Germany). The autoclave underwent natural cooling to room temperature after it was finished. The reddish precipitate was centrifuged and thoroughly cleaned using distilled water three times. The resultant reddish α-Fe_2_O_3_ precipitate was then dried for a total of 12 h at 80 °C.

In order to create n-TiO_2_@α-Fe_2_O_3_, predetermined quantities of n-TiO_2_ nanoparticles and α-Fe_2_O_3_ nanocubes were disseminated in a 60 ml, 95% ethanol solution in a 150 ml round-bottom flask using an ultrasonic treatment for 15 min. The reaction flask was then heated to 60 °C until the solvent had completely evaporated. The mixture was then agitated using a hotplate/stirrer for 24 h. The resultant composite powder particles were sintered in a 120 °C electric oven for two hours before being left at room temperature for additional investigation. The weight ratio of the n-TiO_2_ in the final n-TiO_2_@α-Fe_2_O_3_ nanostructured composite was altered as 1.5 wt%, 2.3 wt%, 4 wt%, and 9 wt% to evaluate the influence of n-TiO_2_ on the catalytic activity. Under visible-light illumination, the catalytic activity of several produced n-TiO_2_@α-Fe_2_O_3_ nanocube photocatalysts for the conversion of methanol into formaldehyde was investigated.

### Characterization techniques

The shape, structure, composition, electronic, and optical properties of nanostructured materials must be shown. The JEM-1230 transmission electron microscope (Joel, Japan) was used to capture TEM pictures, and it was run at 120 kV. Drops of the test material mixed with the appropriate solvent were placed on a C-coated copper grid (Formvar, 300 mesh, Ted Pella) to create samples for TEM, which were then allowed to evaporate in the open air. Using a JEOL JSM 6100 scanning electron microscope set to 5 kV, SEM pictures were captured. A piece of the powder sample was placed on conductive Al tape to create samples for SEM. The stage of a JSM 6100 SEM (JEOL) scanning electron microscope, run at 30 kV, was used to perform the EDAX measurements.

The D8-Discover X-ray diffractometer (Bruker, UK) was used to measure the X-ray diffraction (XRD) with Cu K radiation at 1.54056 Å. At room temperature, the measurement range was 5 to 80 degrees. For 45 min, data was collected in step-scan mode with 0.01° steps from 5 to 80°. Using Vaseline glue, the powder samples were held in place on a glass slide. By comparing the obtained XRD patterns with reference patterns and the built-in database of the X'Pert HighScore-Plus Software, which is based on the Joint Committee on Powder Diffraction Standards-International Center for Diffraction Data (JCPDS-ICCDD) system, the crystal phases of the prepared materials were identified. Sherrer's formula was used to calculate the average crystallite size from diffraction patterns. X-ray photoelectron spectroscopy (XPS) studies were performed in a UHV environment with a PHI VersaProbe III Scanning XPS Microprobe. High resolution pass energy of 20 eV, emission current of 10 mA, and anode HT of 15 kV were maintained as the working parameters continuously. The C1s peak at 284.5 eV has been utilized as a calibration point in order to take the surface charging effect into account.

The Nicolet 6700 FT-IR spectrometer from Thermo Scientific in Madison, USA, was used to perform fourier transform infrared measurements with an accumulation of 16 scans and an 8 cm^−1^ spectral resolution. The LabRAM HR Evolution Raman Spectrometer (HORIBA Scientific, France), which has a 532 nm laser source for excitation, was used to acquire the Raman spectra. With a grating spectral resolution of 300, a 5% neutral density (ND) filter, and an acquisition time of 5 s, the spectrum was acquired using 10 accumulations. The V-630 Spectrophotometer (JASCO, Japan) was used to perform the DRS measurements. In order to record the UV–Vis spectrum based on the diffuse reflectance, the powdered samples were compressed into discs first. A xenon short-arc lamp was used as the exciting source in an LS55 spectrofluorometer (Perkin Elmer, UK) to examine the photoluminescence spectra of diluted samples.

Using the Nova Touch LX2 Pore Size Analyzer (Quantchrome Instruments, USA) at 77 K, nitrogen adsorption and desorption isotherms of various substances were obtained across the range of 0.05 to 1 relative pressure (P/P O). The BET method was used to calculate the specific surface area, and the BJH (Barret-Joyner-Halenda) method was used to estimate the pore size distribution and average pore size from the adsorption branch. All samples were degassed in a N2-He mixture at 100 °C for two hours with a temperature ramp rate of 10 °C min^−1^ before to analytical measurement.

### Photocatalysis measurements

Under visible light illumination, the produced photocatalysts' photocatalytic activity was assessed with respect to the oxidation of methanol in an aqueous solution to simulate it in the real wastewater. UV–visible spectrophotometry was used to investigate the formation of formaldehyde from methanol. The experimental factors including catalyst composition and illumination duration that affect the rate of the photocatalytic oxidation of methanol were investigated.

#### Methanol photo-oxidation experiments

Using a specialized photoreactor, the methanol photocatalytic oxidation studies were carried out. A quartz cell with a volume capacity of approximately 150 ml and two circular flat sides with an inner diameter of 50 mm made up the photoreactor. The front of the solar simulator (Model 67005, Newport, Oriel Instruments, USA), which has an AM1.5 filter, was where the quartz cell was set. The photocatalytic studies were carried out at room temperature with constant 1200 rpm mechanical stirring. In each experiment, a quartz cell was charged with 150 mg of the powdered photocatalyst before 150 ml of an aqueous solution of methanol was added (1% vol. ratio). The quartz cell containing the reaction mixture was subjected to sonication for 10 min, followed by 20 min of incandescent stirring over a mechanical stirrer.

The solar simulator's irradiance was set to 150 mW/cm^2^ for the photooxidation studies, and an 8 cm gap was kept between the edge of the quartz cell's flat surface and the light aperture. In relation to the light source, the quartz cell holding the reaction mixture was lit horizontally. By sampling several aliquots from the illuminated reaction mixture at various exposure durations of 10, 30, 60, 90, 120, 150, and 180 min, the photooxidation of methanol to formaldehyde was tracked. To get rid of the photocatalyst particles, the aliquots were filtered through a 0.2 m syringe filter after being centrifuged for 15 min at 4000 rpm. Using a UV–visible spectrophotometric approach, the amount of methanol that was converted into formaldehyde was measured.

#### Spectrophotometric determination of formaldehyde

The amount of formaldehyde produced as a result of the methanol photo-oxidation was quantified using Nash colorimetric reagent and colorimetric UV–visible spectrophotometry, as described in the literature. The 3,5-diacetyl-1,4-dihydrolutidine, a yellowish chemical, is produced by the reaction of formaldehyde with acetylacetone in the presence of acetic acid in the colorimetric technique. Because it uses a spectrophotometer that is easily accessible, it is quick and sensitive, straightforward to use, and very inexpensive.

In order to make the Nash reagent, 200 ml of distilled water was first mixed with 9.7 × 10^–3^ mol of acetylacetone and 9.7 × 10^–1^ mol of ammonium acetate. Next, 2.5 × 10^–2^ mol of acetic acid were added while agitating the mixture. The combination was first sonicated for 20 min, then diluted to a total volume of 500 ml, and then it was placed to a dark bottle. In the finished mixture, the acetylacetone to ammonium acetate molar ratio was 0.01. An equivalent volume of the centrifuged and filtered reaction mixture was combined with 5 ml of the produced Nash reagent for the colorimetric measurement of formaldehyde. The mixture-containing tube spent 15 min in a warm water bath. A common calibration method based on absorbance at 412 nm was used to calculate the formaldehyde content. Beer- Lambert's law, which links a sample's absorbance to its concentration, was used to build the calibration curve^45^.

### Ethics approval

Not applicable. This manuscript does not involve researching about humans or animals.

### Consent to participate

All of the authors consented to participate in the drafting of this manuscript.

## Results and discussion

### Characterization of n-TiO_2_@α-Fe_2_O_3_ composite photocatalyst

#### Morphological and structural properties

Due to the widespread of these metals and the availability of these metals on earth, metal oxides have attracted significant interest in a variety of applications including catalysis, energy storage, electrochemical applications, photochemical cells, water treatment, and biological applications^46–49^ for their non-toxicity, low cost, and semiconducting properties. Hematite (α-Fe_2_O_3_) is one of these metal oxides and is chemically and thermodynamically stable. It can be easily isolated from the reaction mixture using a magnetic field^50,51^.

**Figure 1:**
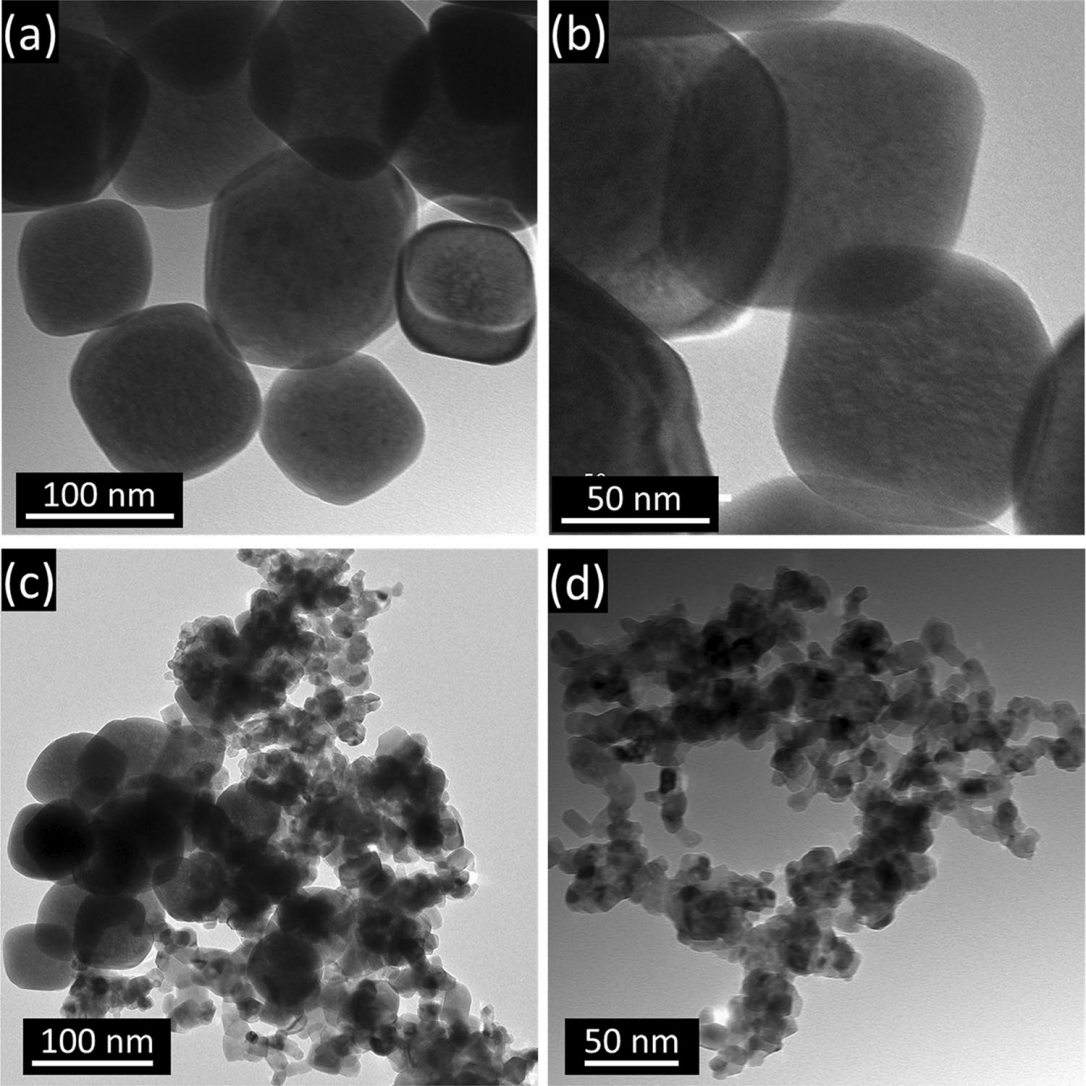
TEM images of (**a**,**b**) α-Fe_2_O_3_ nanocubes and (**c**,**d**) n-TiO_2_@α-Fe_2_O_3_ nanocomposite.

**Figure 2:**
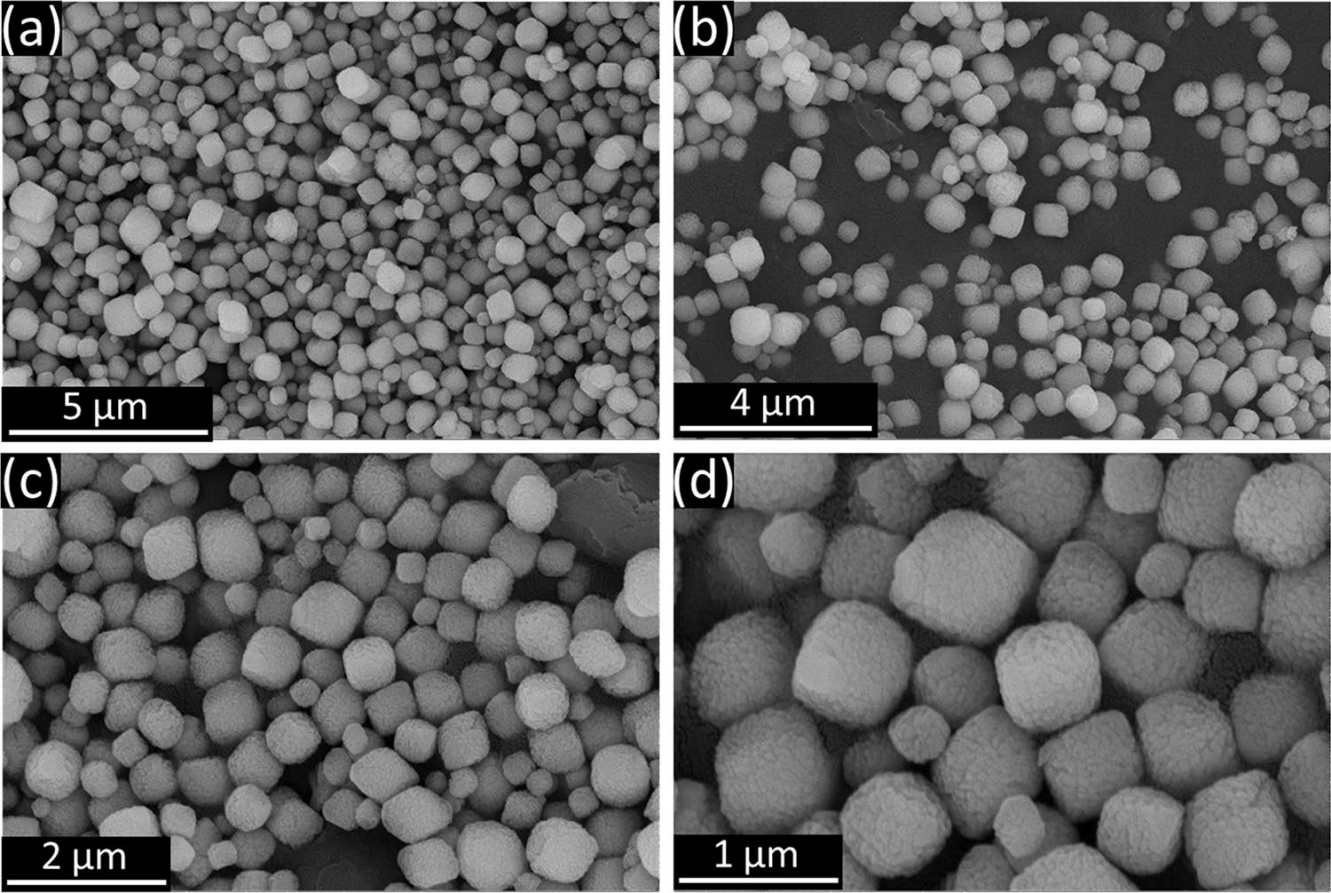
SEM images of α-Fe_2_O_3_ nanocubes.

Hematite (α-Fe_2_O_3_) was depicted in Fig. 1a,b using TEM images. Hematite is a supporting material used to create heterojunctions with nitrogen-doped titania (n-TiO_2_) in order to increase the photocatalytic activity of n-TiO_2_ in the visible spectrum. The photos demonstrated the cubic form and average particle size of 50 nm of the hematite (α-Fe_2_O_3_) nanoparticles. The TEM pictures of the n-TiO_2_@α-Fe_2_O_3_ nanocomposite were displayed in Fig. 1c,d. The surface topography and size distribution of the α-Fe_2_O_3_ NPs were depicted in Fig. 2 using SEM images at various magnification powers; the nanoparticles appear to be cubic in form and have a limited size distribution.

**Figure 3:**
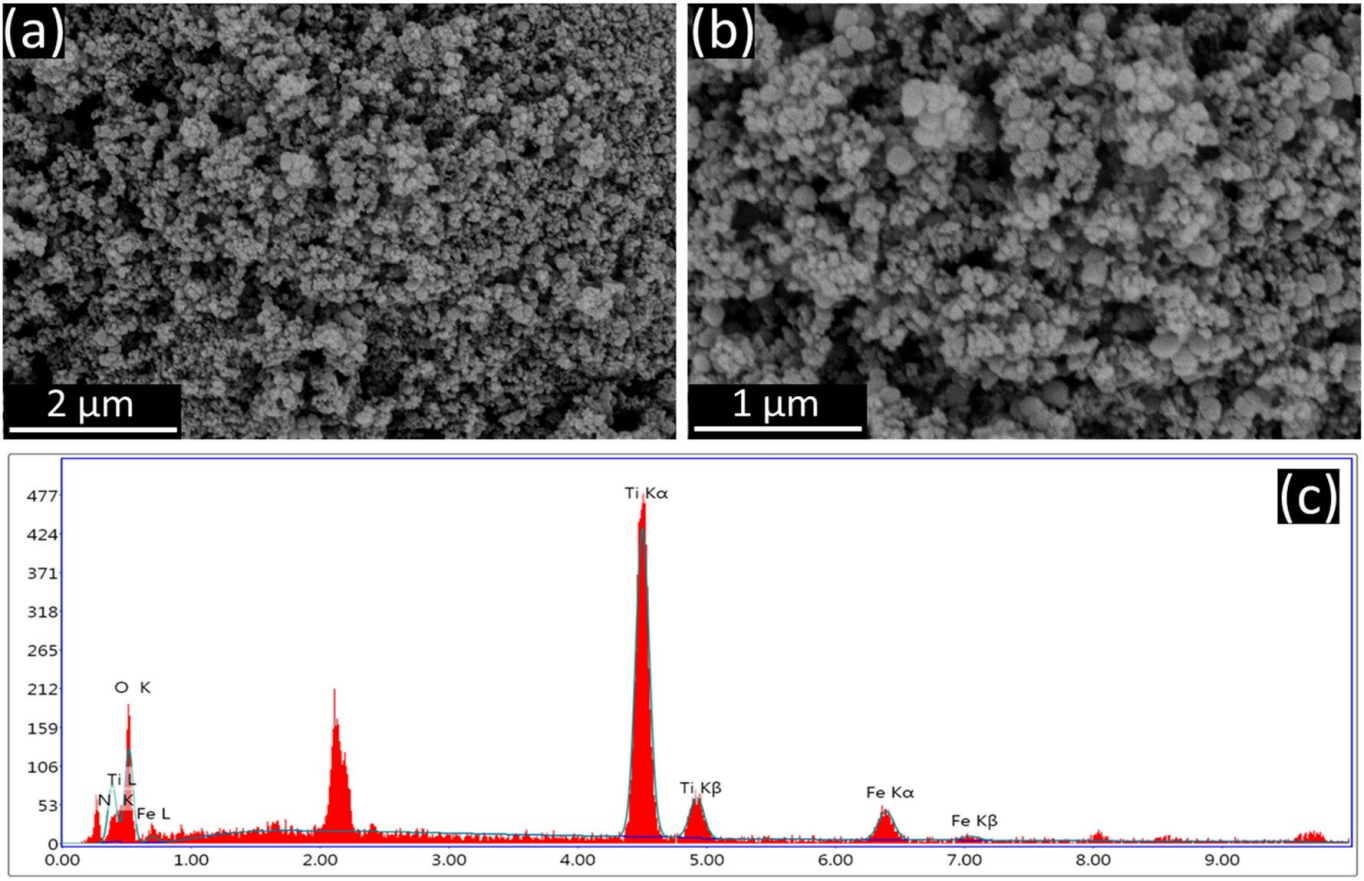
(**a**,**b**) SEM images and (**c**) EDX spectrum of 4%wt n-TiO_2_@α-Fe_2_O_3_.

**Figure 4:**
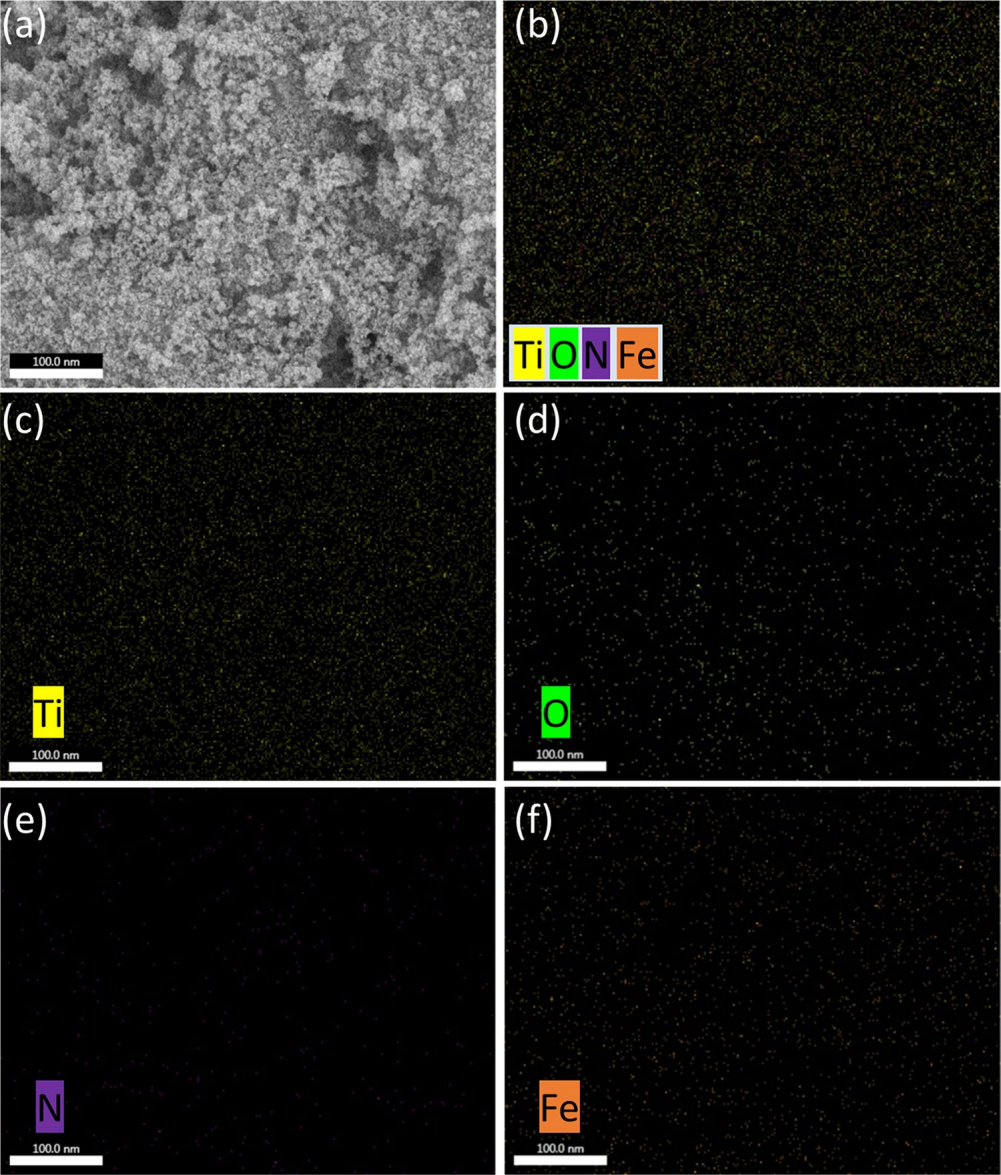
EDX elemental mapping of 4%wt n-TiO_2_@α-Fe_2_O_3_ nanocomposite.

The n-TiO_2_@α-Fe_2_O_3_ nanocomposite's elemental composition was further confirmed by the EDX spectrum, as shown in (Fig. 3c), which demonstrates the existence of Fe, Ti, N, and O peaks. The SEM pictures of the nanocomposite are displayed in Fig. 3a,b. The absence of any further impurity peaks confirms the produced samples' excellent purity and is in line with the XRD and Raman results. On a 4% weight n-TiO_2_@α-Fe_2_O_3_ nanocomposite, energy dispersive X-ray (EDX) analysis was used to map the elements. The elemental mapping of n-TiO_2_ and α-Fe_2_O_3_ in the composite, as seen in (Fig. 4), revealed that nitrogen doped titania was evenly disseminated on the linked supported catalyst, demonstrating the presence of all elements in the composite.

**Figure 5:**
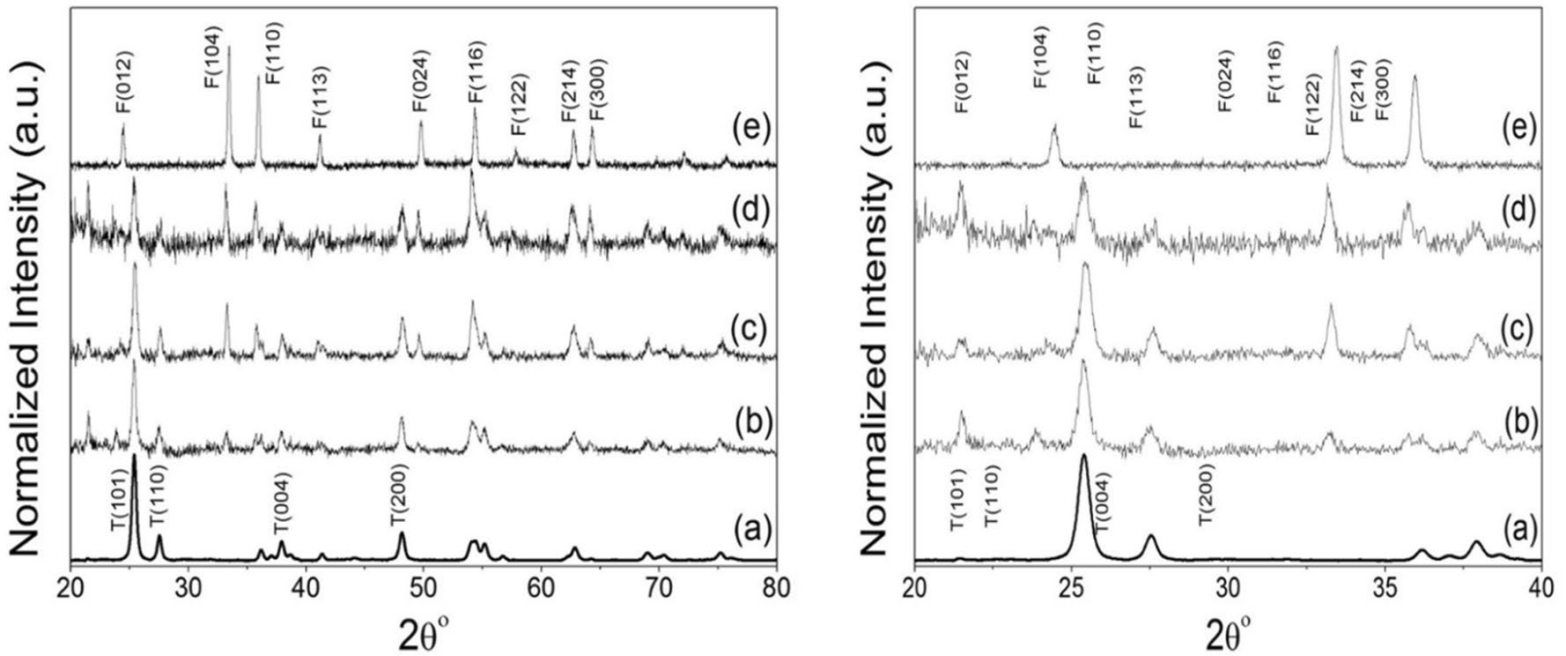
XRD spectra of (**a**) n-TiO_2_, (**b**) 9% n-TiO_2_@α-Fe_2_O_3_, (**c**) 4% n-TiO_2_@α-Fe_2_O_3_, (**d**) 2.3% n-TiO_2_@α-Fe_2_O_3_ and (**e**) α-Fe_2_O_3_ displayed at full scale (left) and enlarged scale (right).

At 2-theta values of 24.3, 33.4, 36, 41, 49.7, 54.2, 57.9, 62.7, and 64.6°, which correspond to (012), (104), (110), (113), (024), (116), (122), and (214) and (300), respectively, the sample of α-Fe_2_O_3_ showed several diffraction peaks typical of the crystalline nanoparticles and attributed to the α-Fe_2_O_3_ rhombohedral phase (matched PDF-01079-007). According to the Scherer equation, the produced α-Fe_2_O_3_ nanoparticles had crystallite sizes of around 40 nm. The previously stated XRD pattern of n-TiO_2_ NPs is depicted in Fig. 5a, which displays numerous diffraction peaks at 2θ values of 25.3, 27.5, and 37.9° for n-TiO_2_ that, respectively, correspond to (101), (110), and (004) crystal planes. The XRD patterns of 9 wt%, 4 wt%, and 2.3 wt% for n-TiO_2_@α-Fe_2_O_3_, respectively, are shown in Fig. 5b–d. They have demonstrated weak intensities for the (101) plane of n-TiO_2_ nanoparticles (2θ = 25.3°) in comparison to that of pure n-TiO_2_, which is consistent with the sample's smaller crystallite size and the evenly dispersed α-Fe_2_O_3_ on n-TiO_2_ surfaces. The photocatalytic activity increases as a result of the size shrinking because a larger surface area is exposed to the photochemical reaction. Additionally, it is possible to see that both n-TiO_2_ and α-Fe_2_O_3_ peaks are present in the three XRD patterns of the three distinct weight percentage composites. These peaks exhibit varying intensities, which indicates that nanocomposites with various weight percentage ratios have formed well. The difference in Fe content between the three composites and the decrease in crystal size are explained by the decrease in intensity of all α-Fe_2_O_3_ planes in the three composites from 2.3 wt% to 4 wt% to 9 wt% of n-TiO_2_@α-Fe_2_O_3_, respectively. This indicates that the incorporation of α-Fe_2_O_3_ with n-TiO_2_ caused the decrease in crystal size, which increased the surface area and increased the photocatalytic activity of the composites. Additionally, the discovery of an overlap between 116 planes of n-TiO_2_ and α-Fe_2_O_3_ confirms the composite's well-formed structure.

#### Electronic properties

**Figure 6:**
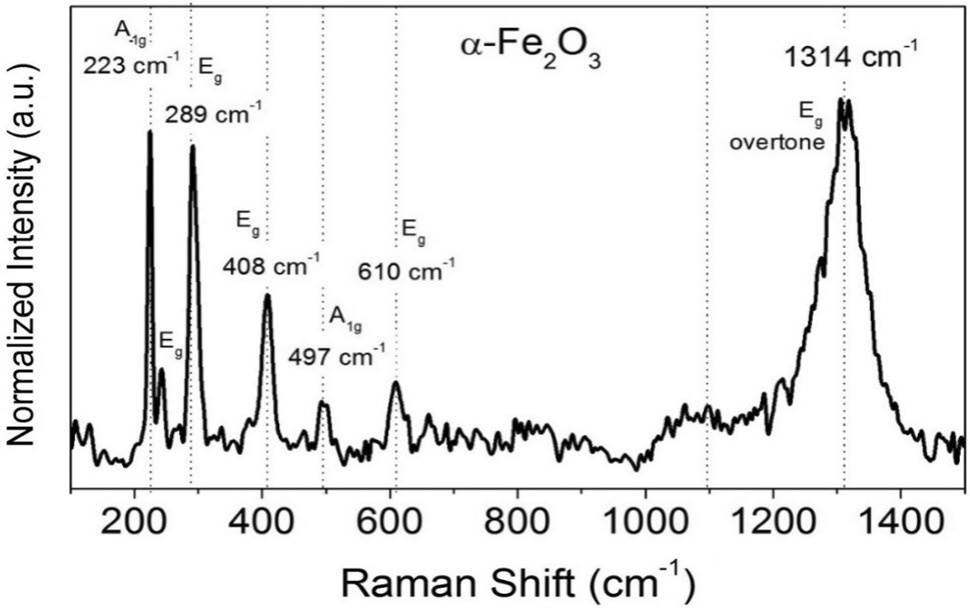
Raman spectrum of α-Fe_2_O_3_ nanocubes.

**Figure 7:**
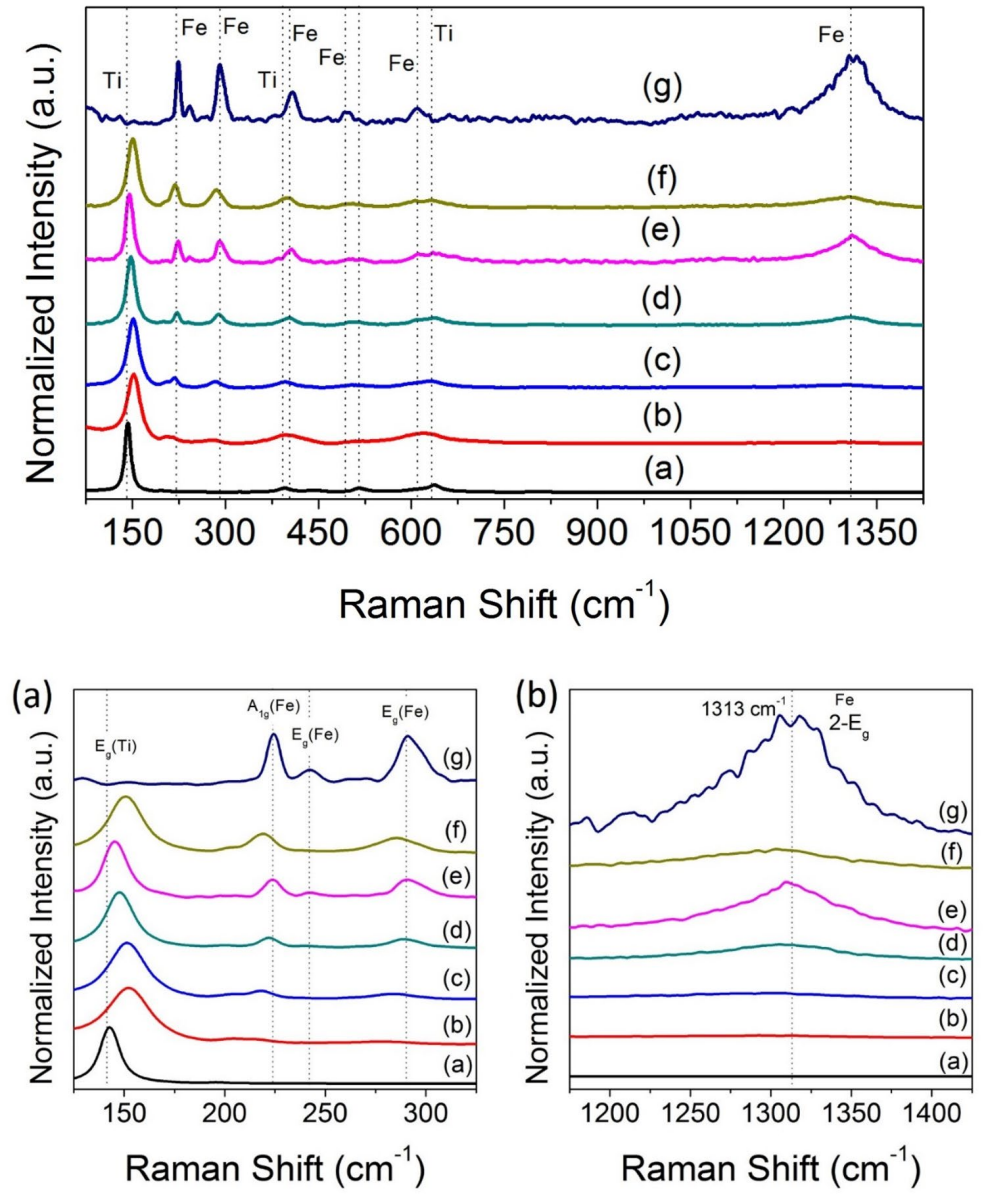
Raman spectrum of (**a**) n-TiO_2_, (**b**) 9% wt n-TiO_2_@α-Fe_2_O_3_, (**c**) 4% wt n-TiO_2_@α-Fe_2_O_3_, (**d**) 2.3% wt n-TiO_2_@α-Fe_2_O_3_, (**e**) 1.5% wt n-TiO_2_@α-Fe_2_O_3_, (**f**) 1% wt n-TiO_2_@α-Fe_2_O_3_, and (**g**) α-Fe_2_O_3_.

In order to investigate the electrical characteristics of hematite (α-Fe_2_O_3_), Raman scattering was used. Figure 6 displays the full-scale Raman spectra (100–1500 cm^−1^) of hematite (α-Fe_2_O_3_) nanocubes, n-TiO_2_ nanoparticles and different n-TiO_2_@α-Fe_2_O_3_ composites. Full-scale representation of the Raman spectrum for α-Fe_2_O_3_ nanocubes (Fig. 6g) revealed that the A_1g_ modes are associated with the peaks centering at 223 cm^−1^ and 497 cm^−1^, while the 2E_g_ modes are associated with the peaks centering at 289 cm^−1^, 408 cm^−1^, and 610 cm^−1^^52^. It had a strong resemblance to literary ideals for rhombohedral crystalline α-Fe_2_O_3_^52^. As shown in Fig. 7g, the peaks were sharp and well-developed, implying that the sample was well-crystallized in accordance with the XRD results. The Raman spectrum of n-TiO_2_ is depicted in Fig. 7a and exhibits peaks at 143 cm^−1^ (E_g_) and 636 cm^−1^ (E_g_) characteristic for anatase phase TiO_2_. The presence of anatase n-TiO_2_ and α-Fe_2_O_3_ peaks in all samples of n-TiO_2_@α-Fe_2_O_3_ in Fig. 7b–f confirms the composites' successful well formation. It is obvious from the figure that the intensity of the peaks related to anatase n-TiO_2_ was decreased in addition to observable broadening that is related to decrease in size of the composite. This decrease in size can lead to an increase in the photocatalyst activity.

#### Surface properties

##### X-Ray photoelectron spectroscopy study

**Figure 8:**
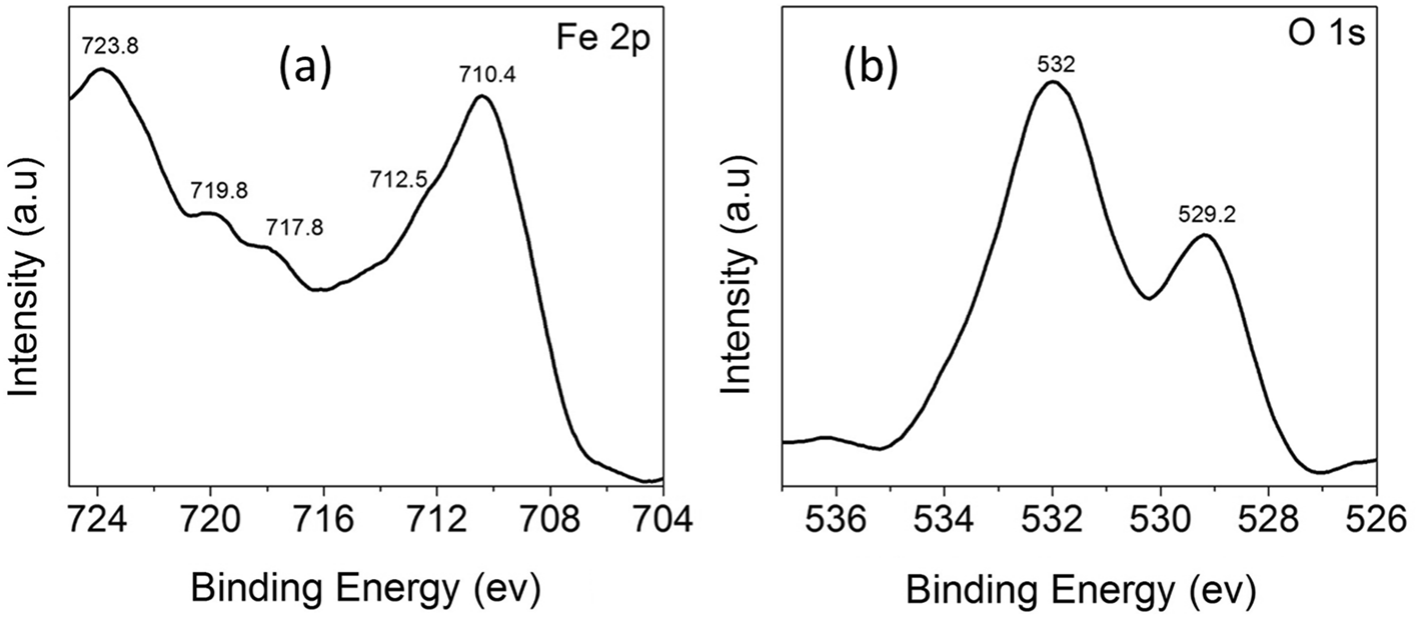
High-Resolution XPS spectra of (**a**) Ti 2p and (**b**) O1s of α-Fe_2_O_3_.

As illustrated in Fig. 8, XPS measurements were carried out to look into the surface flaws and elemental valence states in the α-Fe_2_O_3_ nanocubes. Figure 8a displays the Fe 2p high-resolution XPS spectra that were produced in the 700–730 eV energy range. Two distinctive peaks for Fe 2p_3/2_ and 2p_1/2_ can be seen in this spectrum at energies of 710.4 eV and 723.8 eV, respectively. At binding energies of 712.5 eV and 719.8 eV, these two peaks can be divided into three peaks and assigned to Fe^3+^. Additionally, the BE shows a 14 eV difference between the two peaks, which is typical of Fe^3+^. The peak, however, is exclusive to α-Fe_2_O_3_^51^ and is positioned at 719.8 eV. Two peaks centering at 529.2 eV, which may be attributed to the lattice O^2−^ anions attaching to Fe cations in the Fe–O link, and 532 eV, which corresponds to the absorbed hydroxyl groups, are present in the O 1s peak shown in Fig. 8b^53^. The manufactured powder's iron is in the Fe^+3^ state, according to the XPS results.

**Figure 9:**
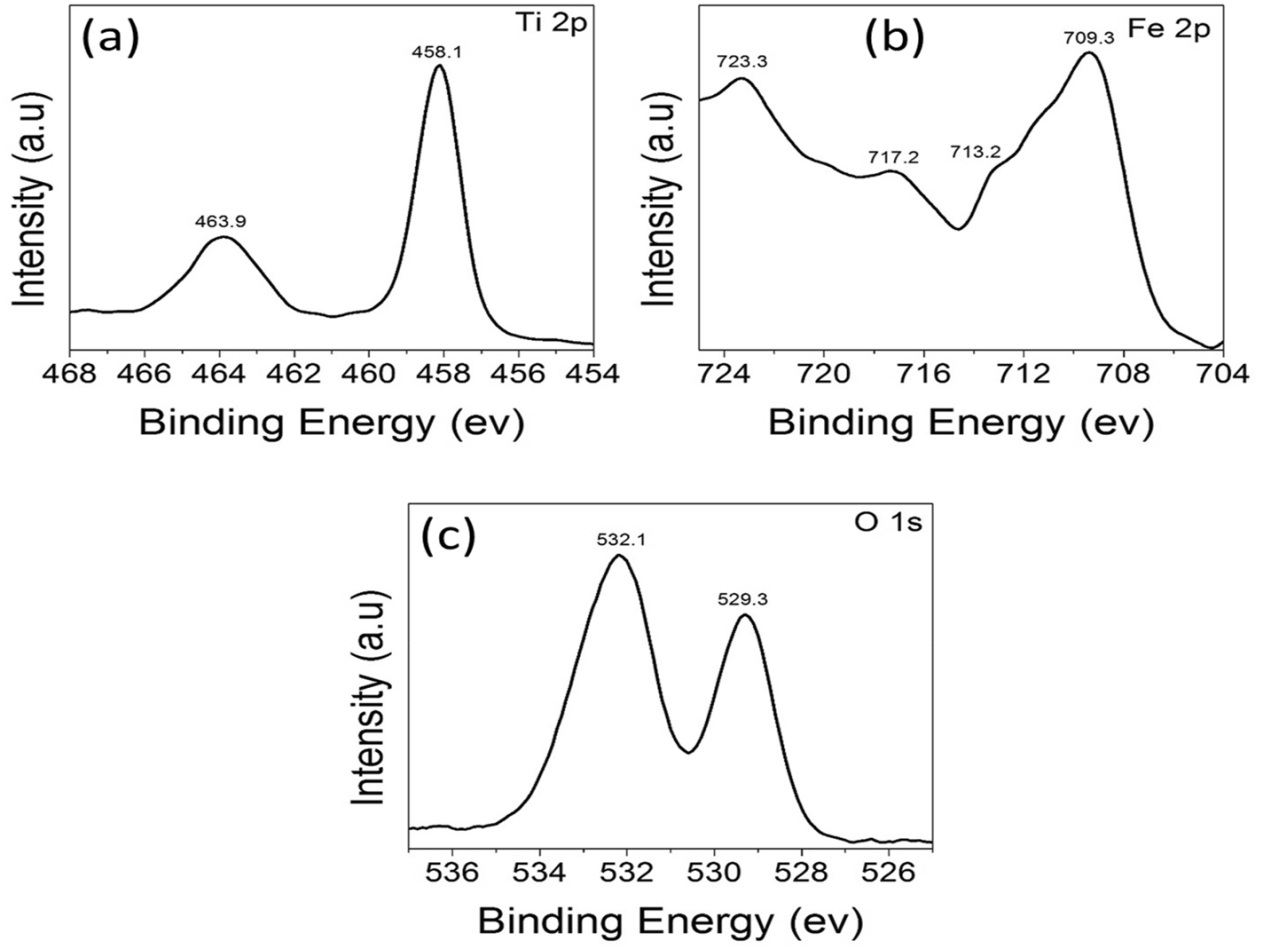
High-Resolution XPS spectra of (**a**) Ti 2p, (**b**) Fe 2p, and (**c**) O1s of 4% wt n-TiO_2_@α-Fe_2_O_3_.

Figure 9's XPS survey scan spectrum of the 4 wt% n-TiO_2_@α-Fe_2_O_3_ composite verifies that Fe 2p, Ti 2p, and O 1s are its principal constituents and provides details on the chemical states of the Fe and Ti elements found there. Two significant characteristic doublets for Ti 2p_3/2_ and Ti 2p_1/2_, with respective binding energies of 458.1 eV and 463.9 eV, can be shown in Fig. 9a for the Ti 2p core level. The two peaks' (BE) separation is 5.8 eV apart, which is typical of Ti^4+^ of the n-TiO_2_. Two distinctive peaks, corresponding to Fe 2p_3/2_ and 2p_1/2_, are shown in Fig. 9b at energies of 709.3 eV and 723.3 eV, respectively. After deconvolution, the two peaks (Fe 2p_3/2_ and 2p_1/2_) can be separated into three peaks at binding energies of 711.2 eV and 725.1 eV, 709.1 eV and 723.5 eV, and 710.5 eV and 725.6 eV, respectively, for Fe^3+^, Fe^2+^, and Fe^4+^. In conclusion, it is clear from the XPS data that the iron in the produced powder is in the Fe^3+^ state, which can interact with n-TiO_2_ lattice. The sharp peak at about 532.1 eV and a second peak at around 529.3 eV may be distinguished from the O 1s peak seen in Fig. 9c. These peaks, which are associated with n-TiO_2_ and α-Fe_2_O_3_, respectively. O^2−^, O^−^, and OH^−^ ions in the oxygen-deficient zones may be responsible for the peak at 529.3 eV and the peak at 532.1 eV, respectively. These peaks may be associated to oxygen in the lattice and O^2−^ ions.

##### BET surface area

Supplementary Fig. S1 shows the nitrogen adsorption–desorption isotherms of TiO_2_, n-TiO_2_, α-Fe_2_O_3_ and 4 wt% n-TiO_2_@α-Fe_2_O_3_ photocatalysts with multipoint BET surface area of 90, 232, 30 and 63 m^2^/g, respectively. The high surface area can be accounted to the accessibility of the redox catalytic activity. The smaller surface area of the composite (63 m^2^/g) compared to pure n-TiO_2_ can be assigned to the occupation of the porous structure with α-Fe_2_O_3_ that exhibit low porosity and hence the overall surface area decreases.

### Photocatalytic activity toward methanol oxidation

#### Methanol conversion to formaldehyde

Supplementary Fig. S2 sketches the elementary steps involved in the charge transfer process in n-TiO_2_@α-Fe_2_O_3_ upon interaction with light. The VB of α-Fe_2_O_3_ is greater than that of n-TiO_2_, and it is thought that the E_v_ at the interface of n-TiO_2_@α-Fe_2_O_3_ is 2.25 eV^54^. According to Supplementary Fig. S2, the optical bandgaps of n-TiO_2_ and α-Fe_2_O_3_ are 3.25 and 2.20 eV, respectively. As a result, it is predicted that the conduction band offset Ec (CBO) at the interface of n-TiO_2_@α-Fe_2_O_3_ is 1.20 eV. The energy band structure of n-TiO_2_@α-Fe_2_O_3_ may be seen in Supplementary Fig. S2 when comparing the band structure of n-TiO_2_ vs. conventional hydrogen electrode (NHE)^54^. Due to its wide band gap, n-TiO_2_ does not exhibit any photo-electronic reaction to visible light. Only α-Fe_2_O_3_ can be stimulated, producing an electron produced by light from its VB to CB. The photogenerated electrons can transfer from CB of α-Fe_2_O_3_ to that of n-TiO_2_ driven by the built-in electric field and the concentration gradient of electrons, while remaining the holes in VB of α-Fe_2_O_3_^54^, due to the aligned equilibrium of the Fermi level at the interface of n-TiO_2_ and α-Fe_2_O_3_, as shown in Supplementary Fig. S2. Thus, as shown by the PL and photocurrent response data in Supplementary Fig. S4, it is possible to increase the separation efficiency of photoinduced electron–hole pairs. The photocatalytic activity of n-TiO_2_@α-Fe_2_O_3_ composite catalysts is a topic covered in a lot of literature^54^. It is generally acknowledged that water and photogenerated holes in VB of α-Fe_2_O_3_ may react to create OH^.^ radicals. Moreover, oxygen and the electrons in CB of n-TiO_2_'s may combine to generate O_2_. These highly active radicals can break down organic compounds into safe substances^49^. It is widely accepted that OH^.^ radicals can be formed via the reaction of water and photogenerated holes in VB of α-Fe_2_O_3_. And electrons in CB of n-TiO_2_ can react with oxygen to form O_2_^−^. These radicals with high activities can degrade organic molecules into harmless substances^54^.

The mechanism of the oxidation of methanol to formaldehyde can be according to Migani, and Blancafort^55^. Photocatalytic OH bond dissociation is relevant in the context of methanol oxidation to formaldehyde CH_2_O^55^. This reaction involves the cleavage of the O–H bond and once C–H bond, resulting in the CH_2_O species adsorbed at a Ti_cus_ site and two O_br_H groups. In the most widely accepted stepwise mechanism of the O–H bond dissociation precedes the C–H bond dissociation. This mechanism goes through the ground state adsorbed ⋅CH_3_O intermediate, and both steps are photoinitiated^55^. CH_2_O is also obtained from across band gap photoexcitation of a pure layer of adsorbed ⋅CH_3_O species, which is formed by coadsorbing CH_3_OH with oxygen atoms^55^. These experiments provide evidence for the ⋅CH_3_O adsorbate being the active species for h mediated oxidation of ⋅CH_3_O on n-TiO_2_ forming formaldehyde^50^. This mechanism is in accordance with previously reported research^56^. According to the earlier reported research, the mechanism of HCHO formation from ⋅CH_3_O is well established and can be described by Eqs. (1) and (2)^17^

1 $${\text{h}}^{ + } \left( {{\text{surface}}} \right) \, + {\text{ CH}}_{{3}} {\text{OH}} \to {\text{ H}}^{ + } + \, \cdot {\text{CH}}_{{3}} {\text{O}}$$

2 $${\text{n-}}{\text{TiO}}_{{2}} + \, \cdot {\text{CH}}_{{3}} {\text{O }} \to {\text{ HCHO }} + {\text{ H}}^{ + } + {\text{ e}}_{{{\text{CB}}}}^{ - }$$

In addition, pure porous WO_3_ was selective mainly towards the oxidation of MeOH to formaldehyde (HCHO, ~ 60%)^10^. This could be due to the formation of hydroxyl radicals (·OH) from the reaction of adsorbed H_2_O with oxidizing h^+^, which could then selectively oxidize MeOH to HCHO. Adsorbed methoxy groups (⋅CH_3_O) could also be present on the catalyst surface after MeOH dissociation, and direct oxidation of these groups^10^ to HCHO through reaction with h^+^ is also expected^10^. Importantly, CO_2_ formation was negligible when WO_3_ was used as a catalyst. This result illustrates the highly selective nature of this catalyst to primarily one oxidation product of MeOH even with a high O_2_ content in the feed. Also, this means that the photocatalytic oxidation of MeOH to HCHO and the desorption of HCHO occurred at a greater rate than the formation of CO_2_. Methyl formate (MF) resulted from the photocatalytic oxidative coupling of HCHO and surface-adsorbed methoxy species that were present after the dissociative adsorption of MeOH^10^. Other theories for photo-oxidation and MF formation have been reported, that are not supported to be happen in our study if so, the catalyst namely WO_3_ was used. This finding is consistent with our findings that the presence of α-Fe_2_O_3_ with n-TiO_2_ make it selective towards formaldehyde formation rather than CO_2_ formation^10^.

**Figure 10:**
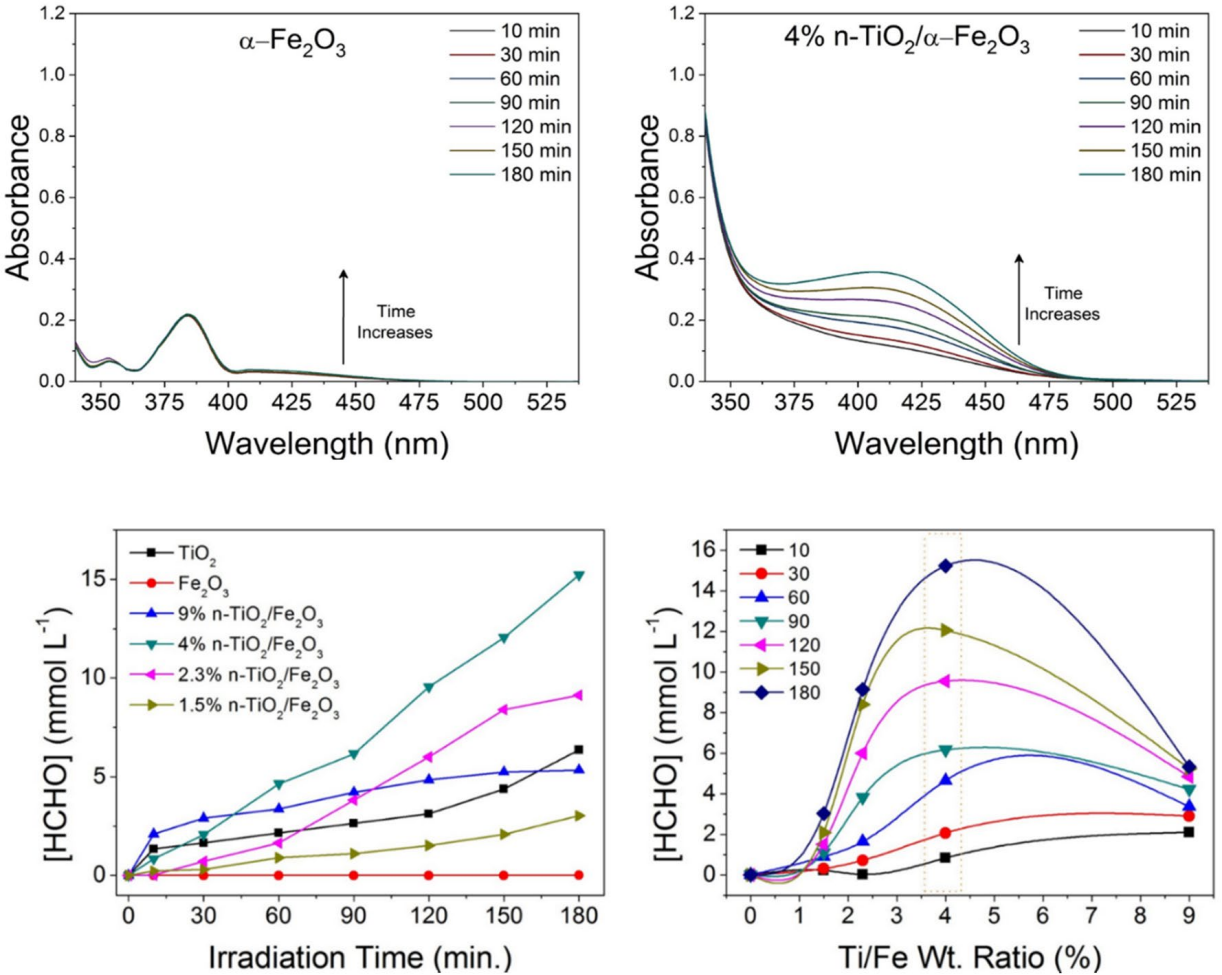
(**Top**) Temporal UV–Visible absorption spectra showing the progressive oxidation of methanol to formaldehyde under visible-light illumination up to 3 h under continuous stirring and exposure to the light stream. (**Bottom-a**) A plot comparing the quantity of formaldehyde generated as a function of irradiation time for TiO_2_, α-Fe_2_O_3,_ 1.5% n-TiO_2_@α-Fe_2_O_3,_ 2.3% n-TiO_2_@α-Fe_2_O_3,_ 4% n-TiO_2_@α-Fe_2_O_3,_ and 4% n-TiO_2_@α-Fe_2_O_3_ showing the improved HCHO formation rates in case of n-TiO_2_. (**Bottom-b**) Plot showing the effect of the composite ratio on the quantity of HCHO formed after visible-light illumination.

Figure 10 (Top) displays the temporal UV–visible absorption spectra that demonstrate the gradual oxidation of methanol to formaldehyde under visible-light illumination for up to 3 h while stirring continuously and being exposed to the light stream. Figure 10 (Bottom a) shows a curve comparing the amount of formaldehyde produced as a function of irradiation duration for n-TiO_2_, α-Fe_2_O_3_, 1.5% n-TiO_2_@α-Fe_2_O_3_, 2.3% n-TiO_2_@α-Fe_2_O_3_, 4% n-TiO_2_@α-Fe_2_O_3_, and 4%n-TiO_2_@α-Fe_2_O_3_ showing the improved HCHO formation rates in case of n-TiO_2_. Additionally, Fig. 10 (Bottom b) highlighted the plot illustrating how the composite ratio affected how much HCHO generated in response to visible-light illumination. The photooxidation was conducted for different illumination times ranged from 10 to 180 min. It is clear that the absorbance increases with time which reflects the increase in methanol conversion to formaldehyde. The increase in the photocatalytic activity of 4 wt% n-TiO_2_@α-Fe_2_O_3_ composite over pure α-Fe_2_O_3_ can be attributed to the increase in the photogenerated carriers numbers within n-TiO_2_ and α-Fe_2_O_3_. The amount of formaldehyde reached about 15.2 mmol L^−1^ in case of 4 wt% n-TiO_2_@α-Fe_2_O_3_ whereas it reached about 9.1, 6.3, 5.3, 3.02 and 0.01 mmol L^−1^ in case of 2.3 wt% n-TiO_2_@α-Fe_2_O_3_, n-TiO_2_, 9 wt% n-TiO_2_@α-Fe_2_O_3_, 1.5 wt% n-TiO_2_@α-Fe_2_O_3_ and pure α-Fe_2_O_3_, respectively.

Compared the previous work that Li et al.^13^, did the photocatalytic oxidation for methanol and reported that photocatalytic methanol oxidation was carried out using four different bismuth-based semiconductors (BBS), Bi_2_WO_6_, Bi_2_MoO_6_, BiOBr and BiVO_4_, at different temperature (5–50 °C) and catalyst dosage (0.2–2.0 g L^−1^) with using initial methanol concentrations ranging from 12.5 to 250 mM. The author reported that the formaldehyde formation rate for all photocatalysts increased as a function of each of these system parameters and he found that Bi_2_WO_6_ had the best formaldehyde formation rate (0.081 mM h^−1^) however, in this study the maximum formed concentration was 15.2 mM h^−1^ after using the proposed nano-materials. Another researcher DePuccio et al.^10^, reported that he succeeded for conversion of methanol to formaldehyde using a different catalyst than this study (Au/Wo_3_) and this confirm our finding that using a novel nanoparticles could enhance the photo catalytic conversion ratio of methanol to formaldehyde.

#### Relationship between optical properties and photocatalytic activity

The two absorption bands at 251 nm and 315 nm, which are connected to the absorption in the UV–Vis region, may be seen in Supplementary Fig. S3 for n-TiO_2_. In the visible region, n-TiO_2_ exhibits very small absorption band at 445 nm as the results of nitrogen doping. However, in the case of the n-TiO_2_@α-Fe_2_O_3_ nanocomposite, in addition to the two bands of n-TiO_2_ that are attributed to the intrinsic absorption band obtained from the bandgap transition of n-TiO_2_ and α-Fe_2_O_3_, respectively, it can be noted the formation of a new absorption band at 517 nm. Additionally, this outcome supported the composite's well-formedness. Comparatively to n-TiO_2_ and n-TiO_2_@α-Fe_2_O_3_, the absorption band for α-Fe_2_O_3_ nanocubes is moved into the visible range. The wavelengths of these absorption bands are 251 nm, 343 nm, 517 nm (which is due to its intrinsic band gap), and 661 nm (these values are typical band edges for α-Fe_2_O_3_). The band gap of the heterostructured n-TiO_2_@α-Fe_2_O_3_ will be reduced after coupling with the narrow band-gap n-TiO_2_@α-Fe_2_O_3_, which will require less energy to separate electron–hole pairs. A higher visible photocatalytic activity could result from increasing the quantity of photogenerated carriers under visible light illumination.

The efficacy of charge carrier trapping, immigration, and transfer is assessed using the photoluminescence emission spectra, which are produced by the recombination of photogenerated carriers (holes and electrons). Lattice flaws and oxygen vacancies are also studied using photoluminescence (PL) spectroscopy. The emission spectra of TiO_2_, n-TiO_2_, and 4 wt% of n-TiO_2_@α-Fe_2_O_3_ are shown in Supplementary Fig. S4 at an excitation wavelength of approximately 320 nm. In the conduction and valance bands of TiO_2_, there is a weak UV shoulder at 384 nm and a minor peak at 421 nm that are attributed to band edge luminescence, which is connected to photoinduced electron–hole recombination. The signal at 480 nm, on the other hand, is a result of a charge transfer transition from a Ti^3+^ to an oxygen anion in a TiO_6_^–8^ complex that is connected to surface oxygen vacancies. The positions of the peaks in the PL spectra of n-TiO_2_ powders agree with those of the undoped TiO_2_, but the intensity of the peaks noticeably decreases, possibly due to excitons trapped at shallow level defects or an electronic transition caused by levels of defect like oxygen vacancies in the band gap. It can be shown that when n-TiO_2_@α-Fe_2_O_3_ is combined with TiO_2_, the peak intensities of the PL spectra are reduced relative to those of TiO_2_ and n-TiO_2_^53^.

Additionally, this leads to the creation of extra energy levels within the band gap of n-TiO_2_, which is what produces the characteristic emission peak at 374 nm that is attributed to Fe^3+^. The lower the PL intensity, the lower the recombination rate, which means that Fe^3+^ ions increase the likelihood of interfacial transfer of photoinduced electrons and holes. The PL spectrum is the result of charge carrier recombination. Additionally, confirm that Fe^3+^ could entrap charge carriers and prevent them from recombining directly, increasing the amount of photogenerated carriers and enhancing the photocatalytic activity of the photocatalyst. According to the PL data, the 4 wt% n-TiO_2_@α-Fe_2_O_3_ photocatalyst has a substantially better photocatalytic activity and a much lower recombination rate for electrons and holes produced by light^56^.

Mesoporous RuO_2_-TiO_2_ nanocomposites at various RuO_2_ concentrations were made via a straightforward sol–gel process in only one step^32^. The test reaction for determining the mesoporous RuO_2_-TiO_2_ nanocomposites' photocatalytic activity under UV and visible light is the photocatalytic CH_3_OH oxidation to HCHO. The amount and degree of RuO_2_ particle loading on the TiO_2_ have a significant impact on the photooxidation of CH_3_OH, indicating the RuO_2_ nanoparticles' unique influence on this visible-light photocatalytic activity. In the instance of visible light^28^, we wanted to improve the photooxidation of CH_3_OH to produce HCHO by altering the TiO_2_ bandgap by adding RuO_2_. This allowed us to absorb and use the visible section of the solar spectrum, which is where the majority of the solar energy is located. HCHO formation would benefit from the addition of 0.5 wt% RuO_2_ to TiO_2_. Consequently, the observed increase in the yield of the photocatalytic methanol oxidation under visible light irradiation28 will be caused by the acceleration through the electron transfer catalysis generated by Ru^4+^ ions.

By photocatalytically oxidizing CH_3_OH to HCHO under direct sunshine irradiation^36^, the photocatalytic activity of P25, P25-500, P25-600, and P25-700 as well as AT was assessed. The semiconductor photocatalyst (TiO_2_) excites the electrons from the valence band (VB) to the conduction band (CB) upon exposure to light energy equal to or higher than the band gap energies (h_t_), resulting in the creation of positive holes (h^+^_vb_) and electrons (e^−^_cb_) in the VB and CB. In the photocatalytic reaction^36^, the photogenerated positive holes form the hydroxyl (⋅OH) radical, a potent oxidizing agent, by reacting with the surface hydroxyl group (^−^OH) or an adsorbed water molecule. The surface-absorbed CH_3_OH and the newly produced, highly potential hydroxyl (⋅OH) radical quickly react to produce HCHO. Moreover, the photogenerated electrons form superoxide radical anion O_2_^⋅^ and H_2_O_2_ when they interact with electron acceptors like O_2_ adsorbed on the catalyst's surface or dissolved in water. Via the intermediary production of the ⋅OH radical^58^, this may also be used to oxidize CH_3_OH to HCHO.

Bi_2_WO_6_, Bi_2_MoO_6_, BiOBr, and BiVO_4_ were used in four distinct photocatalytic methanol oxidation experiments employing bismuth-based semiconductors (BBS) under various system temperatures, bubbling rates, catalyst dosages, and starting methanol concentrations^13^. It was discovered that each of these system characteristics enhanced the formaldehyde generation rate for all photocatalysts. Bi_2_WO_6_ was shown to have the greatest formaldehyde production rate out of these four BBS. On Bi_2_WO_6_, a hypothesized interfacial mechanism for the oxidation of methanol to formaldehyde has been made. It has been shown before^13^ that the generation of formaldehyde results from two subsequent photooxidation processes. The [Bi-O] layer is principally responsible for the reactive sites in semiconductors based on bismuth. A link between the oxygen in the methanol and the bismuth atom may have formed as the methanol adsorbed onto the layer. It's possible that the hydrogen in one methanol's hydroxyl interacted with the hydroxyl of another methanol to produce H_2_O, which then allowed the methyl to make a direct bond with the oxygen in [Bi-O]^13^. This is the initial stage of O–H dissociation-based methoxy formation^55^. The breaking of the C–H bond leads to the subsequent step, the production of formaldehyde. As was previously mentioned, the excited ⋅CH_3_O radicals undergo a C–H bond dissociation to produce formaldehyde^54^, and CH_3_O is an intermediate that has potential hole-trapping sites.

## Conclusion

In conclusion, the solvothermal approach of producing n-TiO_2_@α-Fe_2_O_3_ (which is relatively straightforward, affordable, and scalable) results in metal oxide with a high degree of purity. This is supported by the thorough analysis of the metal oxides and their heterostructured composites with n-TiO_2_. Under 3 h of visible light exposure, the photocatalytic activity of this photocatalyst was studied for the conversion of methanol to formaldehyde. The details of the experimental setup were provided. In order to calculate the concentration of formaldehyde produced by this reaction in terms of absorbance, the spectroscopic monitoring of methanol conversion into formaldehyde was carried out using a colorimetric approach. From this procedure, it may be inferred that n-TiO_2_ absorbs light at a higher rate than pure TiO_2_. Additionally, the results of photoluminescence and diffuse UV-V is reflectance confirmed these absorption findings. A brief description of the varied wt% of n-preparation TiO_2_@α-Fe_2_O_3_ process was given. The development of a heterojunction between n-doped TiO_2_ and α-Fe_2_O_3_ by a specified wt% of 4% and 2.3% for n-TiO_2_@α-Fe_2_O_3_ increases the photocatalytic activity, according to results of the photocatalytic activity. The surface area has no bearing on photocatalytic activity, as shown by the fact that n-TiO_2_@α-Fe_2_O_3_ photocatalyst has 63 m^2^ g^−1^ of surface area, making it more photoactive than n-TiO_2_, that has 232 m^2^ g^−1^. The synthesis of TiO_2_, n-TiO_2_, α-Fe_2_O_3_, and various weight percentage ratios of their nanocomposites was validated through analysis by several characterization techniques (SEM, TEM, EDX, XRD, Raman, FTIR, XPS, BET, DRS, and PL). We can therefore draw the conclusion that the primary determinant is the photocatalyst structure, which is largely dependent on a number of factors, such as band gap energy, band gap position, and the effectiveness of charge carrier formation and separation and these methods for treatment of wastewater containing hazardous materials such as methanol help the researchers to find new technologies for wastewater treatment in the future^57^.

## Supplementary Information

Supplementary Figures.

## Data Availability

The datasets used and/or analyzed during the current study are available from the corresponding author upon reasonable request.
