# Supplementary material for: Fabrication of nitrogen doped TiO2/Fe2O3 nanostructures for photocatalytic oxidation of methanol based wastewater
Source: Sci Rep. 2023 Mar 17;13:4431. doi: 10.1038/s41598-023-31625-5 (PMC10023745; doi:10.1038/s41598-023-31625-5)
Supplement: Supplementary file 1 — Supplementary Figures. [file 41598_2023_31625_MOESM1_ESM.docx]

**
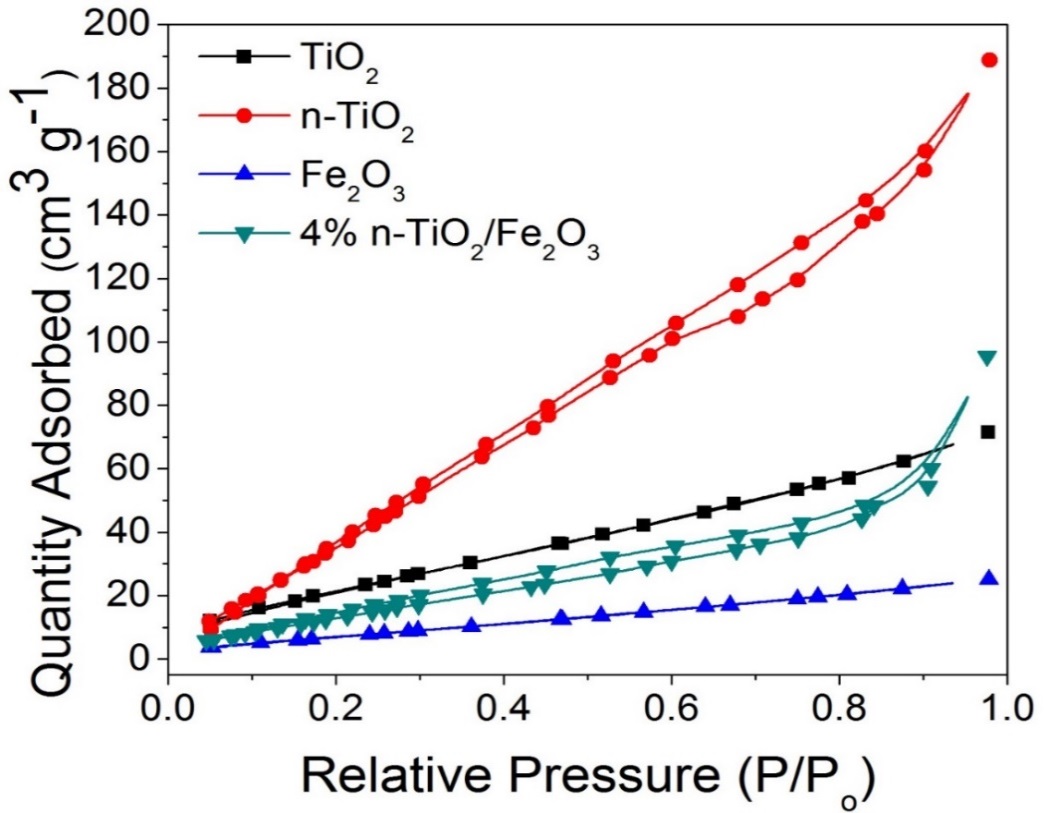
**

**Supplementary Figure S1.** Nitrogen adsorption-desorption isotherms of TiO_2_, n-TiO_2_, α-Fe_2_O_3_ and 4 wt.% n-TiO_2_@α-Fe_2_O_3_ photocatalysts with multipoint BET surface area of 90, 232, 30 and 63 m^2^/g, respectively.


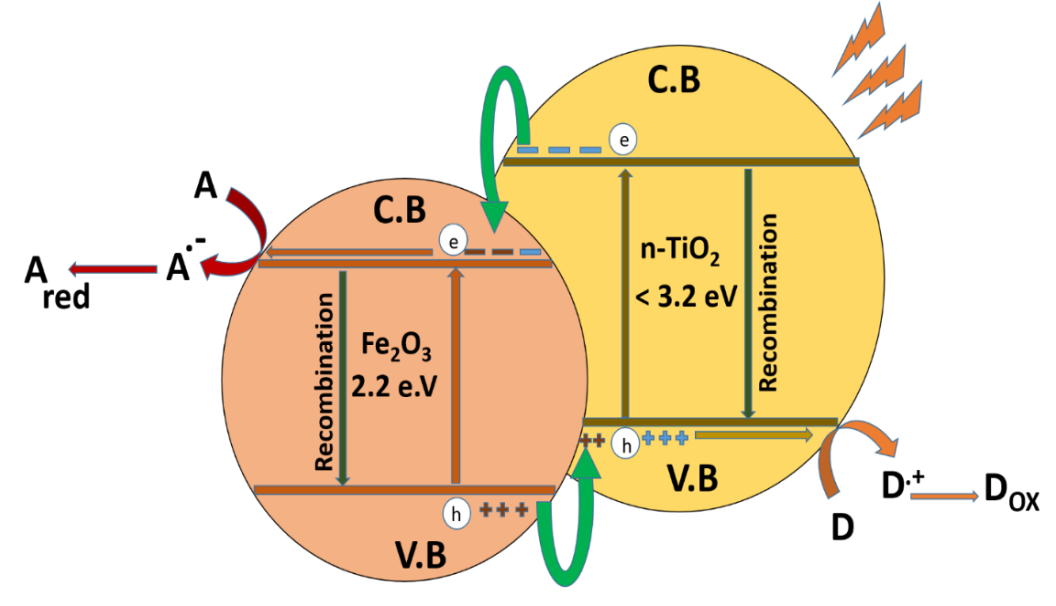


**Supplementary Figure S2.** Sketch of the elementary steps involved in the charge transfer process in n-TiO_2_@α-Fe_2_O_3_ upon interaction with light.


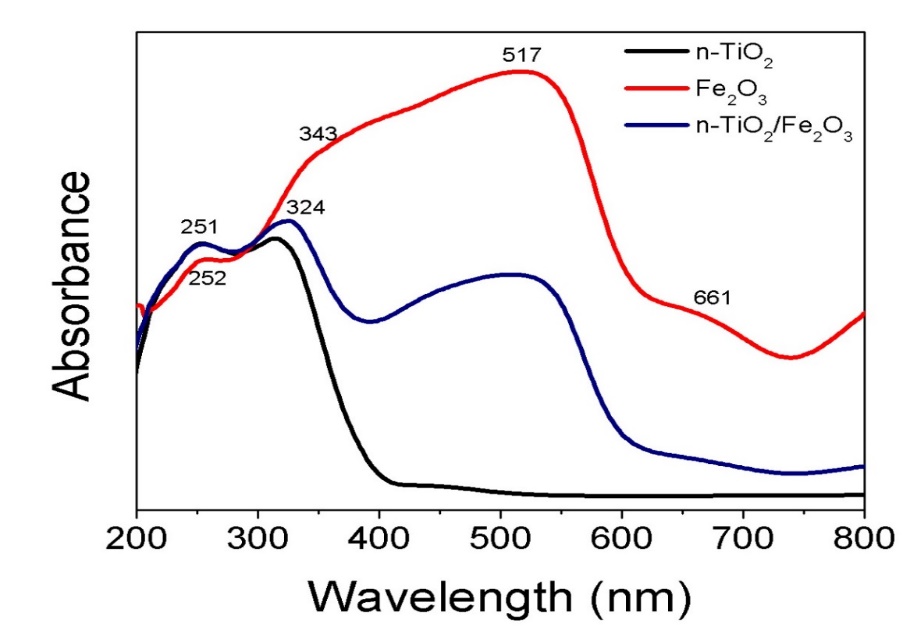


**Supplementary Figure 3.** UV-vis diffuse reﬂectance spectra (DRS) of the n-TiO2 NPs, α-Fe_2_O_3_ NPs, and n-TiO_2_@α-Fe_2_O_3_ nanocomposite.


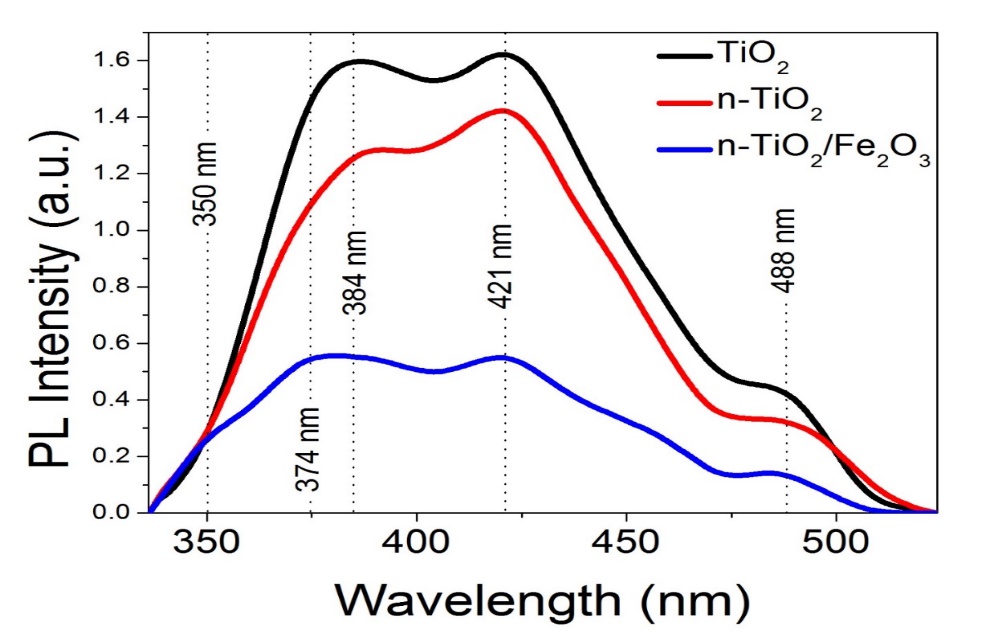


**Supplementary Figure S4.** Photoluminescence (PL) spectra of TiO_2_, n-TiO_2_, and 4 wt.% n-TiO_2_@α-Fe_2_O_3_ photocatalysts in the UV-Vis spectral region of 330-550 .
